# Pharmacokinetics, Pharmacodynamics and Immunogenicity of AC02, a Novel Synthetic Derivate Peptide of Human Adrenocorticotropic Hormone for Infantile Spasms

**DOI:** 10.3390/pharmaceutics18070860

**Published:** 2026-07-14

**Authors:** Shunbo Zhao, Bingda Wu, Hui Shen, Qi Zhou, Minlu Cheng, Chang Shu, Li Ding

**Affiliations:** 1 Department of Pharmaceutical Analysis, School of Pharmacy, China Pharmaceutical University, Nanjing 211198, China; zsb261902@163.com (S.Z.); wubingda0824@163.com (B.W.); imzsts@yeah.net (M.C.); 2 Nanjing Clinical Tech Laboratories Inc., 18 Zhilan Road, Jiangning District, Nanjing 211100, China; shenhui@klt-pharm.com (H.S.); 15255002101@163.com (Q.Z.); 3 Nanjing Jiening Pharmaceutical Technology, 18 Zhilan Road, Jiangning District, Nanjing 211100, China

**Keywords:** AC02, peptide, adrenocorticotropic hormone, pharmacokinetics, pharmacodynamics, immunoassay

## Abstract

**Objectives**: AC02, a novel synthetic peptide derived from ACTH, is being developed as a potential therapeutic alternative to porcine ACTH_1-39_ for use in infantile spasms. **Methods**: The study comprised single-ascending dose cohorts (0.02, 0.04, 0.08, 0.16 mg/kg AC02) and multiple-ascending dose cohorts (0.04, 0.08 mg/kg AC02 daily for 5 days). A separate positive-control arm received porcine ACTH_1-39_ (25 U/day for 5 days). PD effects (free and total cortisol) were compared head-to-head with the positive control. Plasma concentrations of AC02, porcine ACTH_1-39_, free and total cortisol were quantified by validated LC-MS/MS methods, and anti-drug antibody responses were measured using a validated electrochemiluminescence bridging immunoassay. Population PK/PD modeling characterized the concentration–response relationships for free cortisol. **Results**: Across the 0.02–0.16 mg/kg dose range, AC02 exhibited approximately dose-proportional pharmacokinetics and no accumulation after multiple doses. At 0.04 and 0.08 mg/kg, the baseline-corrected effects on free cortisol were generally consistent with those of porcine ACTH_1-39_. Dynamic monitoring of the free cortisol fraction revealed a biphasic pattern across all groups: an early peak followed by a later rise. Total cortisol, by contrast, showed only a monophasic decline, indicating the limitations of total cortisol as a sole PD biomarker. No unexpected safety signals were observed. At 0.04 mg/kg, AC02 demonstrated pharmacodynamic responses comparable to those of marketed ACTH product based on the biologically active component of free cortisol, with favorable safety and pharmacokinetic profiles. **Conclusions**: This first-in-human study demonstrates that AC02 has favorable pharmacokinetic properties, and an acceptable safety profile.

## 1. Introduction

Infantile spasms (West syndrome) is a severe epileptic encephalopathy that typically presents in infants between 3 and 18 months of age [1,2,3]. Due to its rapidly progressive course and substantial risk of long-term neurodevelopmental deficits, timely diagnosis and early therapeutic intervention are critical for improving patient outcomes [4,5,6]. For infantile spasms, adrenocorticotropic hormone (ACTH) remains the standard first-line treatment, given its proven efficacy in reducing seizure frequency and restoring normal electroencephalographic patterns [7,8]. ACTH works by binding to melanocort in receptors on adrenal cortex, triggering the cAMP/protein kinase A signaling pathway and stimulating glucocorticoid production and secretion [9,10]. For clinical use, ACTH is available in two forms: a natural 39-amino-acid peptide extracted from porcine pituitary, and a synthetic analog (tetracosactide or cosyntropin) comprising the N-terminal 1–24 amino acid sequence, which retains the essential biological activity of the endogenous hormone [11,12]. However, the clinical utility of ACTH is substantially limited by high production costs and restricted availability of the natural formulation, underscoring the need for more accessible and cost-effective therapeutic alternatives [13,14].

**Figure 1:**
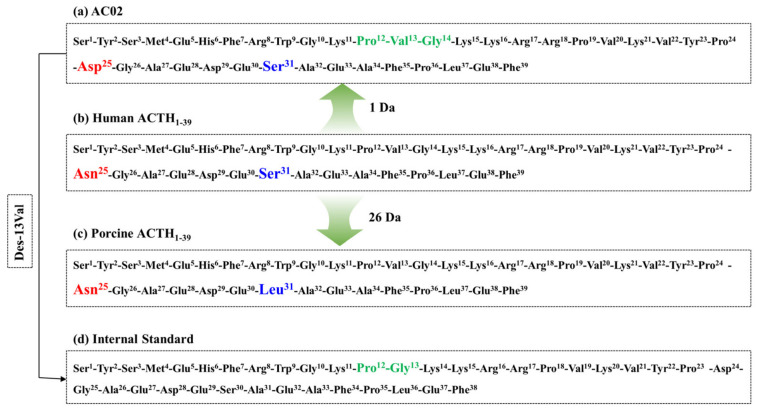
Amino acid sequences of AC02 ((**a**), 4539.24 Da), human ACTH_1-39_ ((**b**), 4538.26 Da), porcine ACTH_1-39_ ((**c**), 4564.31 Da), and Internal Standard Des-13Val ((**d**), 4440.17 Da). The amino acid residue at position 25 (highlighted in red) in AC02 diverges from that in both human and porcine ACTH_1-39_. At position 31 (highlighted in blue), the residue in porcine ACTH_1-39_ differs from those in both AC02 and human ACTH_1-39_. The Des-13Val variant (the internal standard) is generated by deletion of the valine residue at position 13 (highlighted in green) from the AC02 sequence.

To address these limitations, a synthetic ACTH-derived peptide, AC02 (US Patent 11419919B), has been developed. Compared with human ACTH_1-39_, this 39-amino-acid analog contains a single amino acid substitution (Asn^25^ (human ACTH_1-39_) → Asp^25^ (AC02)) (Figure 1) and has demonstrated favorable preclinical pharmacokinetic (PK), pharmacodynamic (PD), and safety profiles. Cortisol, the primary downstream effector hormone of ACTH, circulates in plasma in both protein-bound and free forms; the unbound fraction represents the biologically active component [15]. Long-term exposure to high-dose exogenous corticosteroids or elevated endogenous cortisol is associated with significant metabolic and physiological adverse effects [16]. Consequently, expert consensus and clinical guidelines increasingly recommend minimizing corticosteroid use, adhering to the lowest effective doses, and limiting treatment duration. While total cortisol is commonly measured as a PD biomarker, it is the free cortisol that directly engages with glucocorticoid receptors and mediates both therapeutic and adverse effects [17,18,19]. However, the kinetics of free cortisol following ACTH stimulation remain poorly characterized, particularly its relationship to total cortisol and its potential fluctuations. Understanding these dynamics may provide deeper insight into the time course of drug action and help refine PD assessments for ACTH-based therapies. Therefore, a comprehensive evaluation of AC02 should not only characterize its PK properties but also rigorously assess PD endpoints, especially free cortisol dynamics, in relation to potential safety implications.

PK/PD modeling has become an indispensable tool in drug development, offering a quantitative framework to bridge drug exposure and therapeutic effect [20,21]. The PK profile of ACTH is characterized by a short half-life [22], whereas the PD response, which mediated by downstream steroidogenesis, is noticeably prolonged. This difference creates a hysteresis loop, a phenomenon typically described by an indirect response model, in which plasma concentrations of ACTH decline rapidly while the pharmacological effect endures. Thus, establishing a PK/PD model is essential to accurately capture the intricate exposure-response relationship inherent to such molecules.

The first-in-human study aimed to characterize the pharmacokinetics of AC02 and compare its PD effects (free/total cortisol) with those of the positive control (porcine ACTH_1-39_) in healthy volunteers. Safety and immunogenicity were also evaluated. These findings provide a quantitative PK/PD framework to support the further clinical development of AC02 as a therapeutic alternative to porcine ACTH_1-39_.

## 2. Materials and Methods

### 2.1. Chemicals and Reagents

AC02 (97.7%), human ACTH_1-39_ (99.0%), and rabbit anti-AC02 antibody (0.803 mg/mL) were obtained from Nanjing Hanxin Pharmaceutical Technology Co., Ltd. (Nanjing, China). Porcine ACTH_1-39_ (97.9%) and the internal standard Des-13Val (96.02%) were sourced from Synpeptide Co., Ltd. (Nanjing, China). Biotin-labeled and ruthenium-labeled AC02 were prepared by the authors. Cortisol (99.1%) and its internal standard, cortisol-d4 (97.9%), were purchased from Beijing Manhattan Biotechnology Co., Ltd. (Beijing, China) and SHANGHAI ZZBIO Co., Ltd. (Shanghai, China), respectively. HPLC-grade methanol, acetonitrile, ammonium hydroxide, and isopropanol were obtained from Merck KGaA (Darmstadt, Germany), while formic acid (FA) and human serum albumin (HSA) were sourced from Sigma-Aldrich (St. Louis, MO, USA). Phosphate-buffered saline (PBS) was purchased from Absin Bioscience Inc. (Shanghai, China). The Adrenocorticotropine for Injection (active component: porcine ACTH_1-39_) was provided by Shanghai First Biochemical Pharmaceutical Co., Ltd. (Shanghai, China). Amicon^®^Ultra-0.5 mL centrifugal filters (30 kDa, Merck Millipore, Burlington, MA, USA) were used for plasma ultrafiltration in free cortisol analysis. Drug-free plasma was provided by the Women & Children’s Hospital of Hunan (Changsha, China) (Ethics Approval Number: FE-GCP-LS-2024-022-T). Ultrafiltrate was prepared by centrifuging drug-free plasma at 10,000× *g* for 15 min using a 30 kDa Amicon^®^ Ultra filter.

### 2.2. Study Design

**Figure 2:**
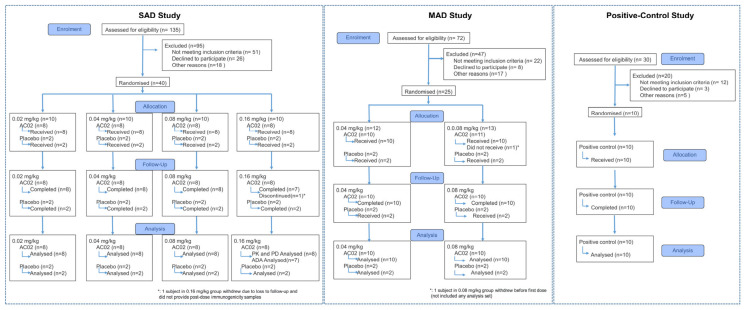
The flow diagram of SAD, MAD and positive-control study.

A single-center, randomized, double-blind, placebo- and positive-controlled Phase 1 trial was conducted in healthy volunteers. The study comprised single-ascending dose (SAD) and multiple-ascending dose (MAD) phases, with a separate positive-control arm receiving porcine ACTH_1-39_ (Figure 2). The trial was conducted at Shandong Provincial Qianfoshan Hospital (Jinan, China) in accordance with the Declaration of Helsinki and approved by the independent institutional ethics committee. Written informed consent was obtained from all participants prior to enrollment. The randomized clinical trial was reported in accordance with the CONSORT 2010 Statement.

A total of 74 healthy volunteers aged 18–45 years with a body mass index of 19–26 kg/m^2^ were enrolled. All participants underwent comprehensive screening, including medical history, physical examination, 12-lead electrocardiography, and standard laboratory tests, to confirm good health. Exclusion criteria included clinically significant medical conditions, smoking history, or documented substance abuse (alcohol or drugs). Participants were required to be medication-free for at least two weeks prior to study entry.

#### 2.2.1. SAD Study

The SAD study evaluated four ascending dose cohorts: 0.02, 0.04, 0.08, and 0.16 mg/kg. In each cohort, 10 participants were randomized to receive a 2 h intravenous infusion of AC02 (n = 8) or placebo (n = 2). Blood samples (4 mL) for PK analysis were collected into K_2_EDTA tubes at pre-dose; at 15, 30, 60, and 90 min during infusion; at the end of infusion (0 h); and at 5, 10, 15, 30 min and 1, 2, 3, 4, 6, and 12 h post-infusion. For PD analysis of free and total cortisol, blood samples (3 mL) were collected on Day −1 (baseline) and Day 1 (treatment day) at pre-dose; at 30, 60, and 90 min during infusion; at the end of infusion (0 h); and at 1, 2, 4, 6, 8, 10, 12, 14, and 24 h post-infusion. The 24 h sample was collected only on Day 1. Immunogenicity samples (3 mL) were collected at pre-dose (Day 1) and on Days 14 and 28 post-infusion.

#### 2.2.2. MAD Study

The MAD study evaluated two dose cohorts (0.04 and 0.08 mg/kg). In each cohort, 12 participants were randomized (10:2) to receive AC02 or placebo as 2 h daily infusions for five consecutive days. PK samples were collected on Days 1 and 5 at the same time points as in the SAD study, with additional pre-dose trough samples on Days 3 and 4. PD samples were collected on Day −1 (baseline) using the same schedule as in the SAD study. On treatment days, PD samples were collected at pre-dose; at 30, 60, and 90 min during infusion; at the end of infusion (0 h); and at 1, 2, 4, 6, 8, 10, 12, and 24 h post-infusion on Days 1 and 5 (the 24 h sample was collected only on Day 5). Additional pre-dose trough samples were collected on Days 2 and 3. Immunogenicity sample collection followed the same schedule as the SAD study.

#### 2.2.3. Positive-Control Study

A cohort of ten participants received 2 h intravenous infusions of Adrenocorticotropine for Injection (active pharmaceutical ingredient: porcine ACTH_1-39_) daily. The dose was 25 U per day for five consecutive days, tapered to 12.5 U on Day 6 and 6.25 U on Day 7, with continuous safety monitoring through Day 9. For PK analysis, blood samples were collected on Days 1 and 5 at pre-dose; at 30, 60, and 90 min during infusion; at the end of infusion (0 h); and at 5, 10, 15, 30 min and 1, 2, 3, 4, 6, and 12 h post-infusion. Additional pre-dose trough samples were collected on Days 3 and 4. On the day before dosing (Day −1), plasma samples were collected at the same time points as on Day 1 to determine baseline concentrations of endogenous human ACTH_1-39_. PD sample collection followed the same schedule as in the MAD study.

#### 2.2.4. Sample Processing

Plasma samples for PK and PD analysis were collected in K_2_EDTA tubes and immediately placed on wet ice; serum samples for immunogenicity analysis were maintained at ambient temperature until processing. All samples were centrifuged within 1 h of collection at 1700× *g* for 10 min at 4 °C and stored at −70 °C until analysis.

### 2.3. Analytical Methods

Plasma concentrations of AC02, porcine ACTH_1-39_, free cortisol, and total cortisol were quantified using validated LC-MS/MS methods according to ICH M10 guideline [23]. Calibration range and mass spectrum parameters of AC02, porcine ACTH_1-39_, free and total cortisol are summarized in Supplemental Table S1. Anti-AC02 antibodies in serum samples were measured by electrochemiluminescence (ECL)-based bridging immunoassay, with anti-drug antibody (ADA) responses evaluated using a multi-tiered strategy consistent with FDA guidance [24].

#### 2.3.1. AC02

AC02 was quantified using a SCIEX Exion LC system coupled with a SCIEX Triple Quad™ 6500^+^ mass spectrometer (Framingham, MA, USA). Chromatographic separation was performed on an ACQUITY UPLC^®^ CSH™ C18 column (50 × 2.1 mm, 1.7 μm; Waters, Milford, MA, USA) with mobile phase A (0.2% formic acid in water) and B (0.2% formic acid in acetonitrile). The gradient elution program was: 0–0.6 min, 15% B; 0.6–1.5 min, linear increase to 20% B; 1.5–2.5 min, hold at 20% B; 2.5–3.0 min, linear increase to 90% B; 3.0–3.9 min, hold at 90% B; 3.9–4.0 min, return to 15% B; 4.0–5.0 min, re-equilibrate at 15% B. The flow rate was 0.3 mL/min. For sample preparation, 50.0 µL plasma was mixed with 25.0 µL internal standard working solution and acidified with 25.0 µL 0.1% formic acid in water, followed by protein precipitation with 250 µL methanol–acetonitrile (1:1, *v*/*v*). After centrifugation, 150 µL supernatant was diluted with 150 µL 0.1% formic acid in water and analyzed by LC-MS/MS.

#### 2.3.2. Porcine ACTH_1-39_

LC-MS/MS conditions were identical to those for AC02. Sample preparation involved protein precipitation followed by micro-solid-phase extraction (μSPE). 200 μL plasma was mixed with internal standard working solution and precipitated with acetonitrile-water (4:1, *v*/*v*). After centrifugation, the supernatant was loaded onto a Oasis^®^ MAX μElution Plate (Waters, Milford, MA, USA) preconditioned with methanol and 5% ammonium hydroxide solution. The cartridge was washed with 5% ammonium hydroxide solution and acetonitrile-water (1:4, *v*/*v*), and analytes were eluted with 5% formic acid in water-methanol (3:7, *v*/*v*). The eluate was diluted with water and analyzed.

#### 2.3.3. Free Cortisol

Free cortisol was analyzed using the same LC-MS/MS system as described for AC02. Chromatographic separation was performed on an ACE Excel 3 Super C18 column (50 × 2.1 mm, 3 μm; ACE, Aberdeen, UK) with mobile phase A (0.1% formic acid in water) and B (acetonitrile). The gradient was: 0–1.3 min, 34% B; 1.3–1.4 min, linear increase to 100% B; 1.4–2.2 min, hold at 100% B; 2.2–2.3 min, return to 34% B; 2.3–3.1 min, re-equilibrate at 34% B. The flow rate was 0.4 mL/min. Free cortisol was isolated by ultrafiltration using Amicon^®^ Ultra−0.5 mL centrifugal filters (30 kDa). The filter was pre-activated with water, equilibrated with drug-free plasma, and centrifuged to collect ultrafiltrate. For protein precipitation, 50.0 μL ultrafiltrate was mixed with internal standard (cortisol-d4, 40.0 ng/mL) and acetonitrile. After centrifugation, 50.0 μL supernatant was diluted with 150 μL 0.1% formic acid in water for analysis.

#### 2.3.4. Total Cortisol

Total cortisol was analyzed using the same LC-MS/MS system and chromatographic conditions as described for free cortisol. For sample preparation, plasma sample (50.0 µL) was mixed with internal standard working solution (cortisol-d4, 25.0 µL, 200 ng/mL) and precipitated with acetonitrile (200 µL) in a 96-well plate. After vortexing and centrifugation (2200× *g*, 10 min, 15 °C), 50.0 µL of the supernatant was diluted with 200 µL 0.1% formic acid in water and analyzed by LC-MS/MS.

#### 2.3.5. Immunogenicity

Anti-AC02 antibodies in human serum were measured using an ECL-based bridging immunoassay. Biotin-labeled and ruthenium-labeled AC02 were used for capture and detection. For sample analysis, serum samples (20.0 μL) were acidified with 20.0 μL of 300 mM acetic acid to dissociate AC02 bound to ADAs. After acidification, 10.0 µL of neutralization buffer (Trizma alkali solution, pH 8.5) and 130 µL of Master Mix solution (containing biotin-labeled and ruthenium-labeled AC02 diluted in assay buffer) were added to each well, and the plate was incubated at room temperature for 1.5 h. Subsequently, 50.0 μL of the mixture was transferred to the pre-blocked Meso Scale Discovery (MSD) streptavidin plates. After the addition of 100 μL of MSD 2X Read Buffer, ECL signals were measured using a MESO QuickPlex SQ 120 analyzer (Rockville, MD, USA). The method was validated according to regulatory guidance, with a minimum required dilution (MRD) of 1:9. Assay sensitivity was 3.77 ng/mL for the screening assay and 6.92 ng/mL for the confirmatory assay. Drug tolerance testing demonstrated reliable ADA detection at AC02 concentrations of 1.0, 5.0, and 10.0 μg/mL using positive controls at 12, 500, and 2000 ng/mL, respectively. A multi-tiered strategy was adopted for ADA determination. For biological samples with ADA-positive results in the confirmatory assay were further evaluated for cross-reactivity with human ACTH_1-39_.

### 2.4. Pharmacokinetic and Pharmacodynamic Analysis

AC02 is a synthetic analog of human ACTH_1-39_ with a single amino acid substitution (Asn^25^ → Asp^25^), resulting in a molecular weight difference of only 1 Da. Due to this structural similarity, chromatographic separation and mass spectrometric discrimination between AC02 and endogenous human ACTH_1-39_ are technically challenging. To address this, baseline plasma samples were collected on Day −1 in the positive-control study to quantify endogenous ACTH_1-39_ concentrations and assess potential interference with post-dose sample quantification. Endogenous human ACTH concentrations in the positive-control study remained below 100 pg/mL, consistent with reported physiological ranges (6–63 pg/mL) [25]. In contrast, post-administration plasma concentrations of AC02 were substantially higher (*C*_max_ > 40 ng/mL), exceeding endogenous levels by more than 400-fold. These findings confirm that endogenous ACTH_1-39_ does not interfere with the quantification of post-dose samples, obviating the need for baseline correction. PK and PD analyses were performed using Phoenix WinNonlin version 8.2 (Certara, Princeton, NJ, USA). PK parameters were derived via non-compartmental analysis. Dose proportionality of AC02 was assessed for the primary PK endpoints: maximum plasma concentration (*C*_max_), area under the plasma concentration-time curve from time zero to the last measurable concentration (*AUC*_0–t_), and area under the plasma concentration-time curve from time zero to infinity (*AUC*_inf_). A power model was applied:

*y* = *α* × dose^*^β^* (1)

where *y* represents the PK variable, *α* is the proportionality constant, and *β* is the exponent. The dose-normalized geometric mean ratio (Rdnm) was calculated as

Rdnm = *r*^^(*β*−1)^ (2)

where *r* is the dose range multiplier (ratio of the highest to lowest dose). Dose proportionality was concluded when the 90% confidence interval (CI) for *β* contained 1 and the 90% CI for Rdnm was entirely within the equivalence range of 0.8–1.25.

For PD analysis of free and total cortisol, the maximum effect (*E*_max_), area under the effect curve from time zero to the last measurable time point (*AUEC*_0–t_), and area under the effect curve from time zero to infinity (*AUEC*_inf_) were determined using non-compartmental analysis. Baseline-corrected parameters (Δ*E*_max_, Δ*AUEC*_0–t_, and Δ*AUEC*_inf_) were also evaluated by subtracting time-matched baseline (Day −1) values from the corresponding values on Day 1 and Day 5 for each treatment. Any negative values resulting from this correction were set to zero.

### 2.5. Population PK/PD Modeling

A population PK/PD model was developed using the PK and free cortisol data from the SAD and MAD studies. Population modeling was conducted using nonlinear mixed-effects modeling implemented in Monolix 24R1 (Lixoft, Antony, France), with parameter estimation via the Stochastic Approximation Expectation-Maximization (SAEM) algorithm. The PK of AC02 was described by a one-compartment model with first-order elimination. As the pharmacological effect is mediated by free cortisol, the temporal response of free cortisol was linked to AC02 plasma concentrations through an indirect stimulation model. The change in baseline-corrected free cortisol (Δfree) over time was described by the following differential equation:

(3) $$\frac{d\Delta free\;cortisol\;levels}{dt}=K_{in}\times \left[\frac{E_{max}^{\prime }\times \;C^{\gamma }(t)}{{EC}_{50}^{\gamma }+C^{\gamma }(t)}\right]-K_{out}\times \Delta free$$

where Δfree is the baseline-corrected free cortisol concentration; *K*_in_ is the secretion rate of free cortisol; *K*_out_ is the first-order degradation rate constant of free cortisol; *C*(*t*) is the predicted AC02 concentration at time t; *E*_max_ is the maximum stimulatory effect; *EC*_50_ is the AC02 concentration to induce 50% of maximum free cortisol; and γ is the Hill coefficient (fixed to 1 in the model). Model performance was evaluated using standard goodness-of-fit diagnostics, including plots of observed vs. population-predicted and observed vs. individual-predicted concentrations. Formal model qualification methods (e.g., visual predictive checks, bootstrap) were not performed, as this exploratory model is intended primarily for descriptive characterization of the concentration–effect relationship in this first-in-human study; predictive performance will be evaluated in future studies with larger sample sizes.

## 3. Results

### 3.1. Study Population

A total of 74 healthy volunteers were enrolled in this study. Demographic characteristics are presented in Table S2. All participants were included in the PK, PD, and immunogenicity evaluations. The study population consisted of 61 male and 13 female participants, aged 18 to 44 years, with a body mass index ranging from 18.9 to 25.8 kg/m^2^. All participants met the eligibility criteria and were included in the pharmacokinetic, pharmacodynamic, and immunogenicity analyses.

### 3.2. Pharmacokinetics

#### 3.2.1. Single-Ascending Dose Study

**Figure 3:**
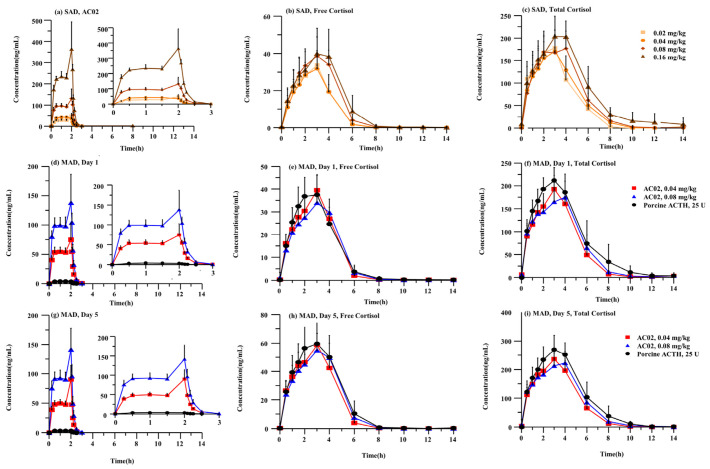
Mean plasma concentration-time profiles following (**a**–**c**) single-ascending dose (SAD) and (**d**–**i**) multiple-ascending dose (MAD) administrations. AC02 (**a**,**d**,**g**); ΔFree cortisol (**b**,**e**,**h**); ΔTotal cortisol (**c**,**f**,**i**). Day 1 data are presented in panels (**a**–**c**) (SAD) and (**d**–**f**) (MAD), while Day 5 data are shown in panels (**g**–**i**) (MAD). The insets in (**a**,**d**,**g**) show magnified views of 0–3 h timepoints, where AC02 concentrations were below the LLOQ after 3 h. Δ values represent baseline-corrected concentrations (Day 1/Day 5 values minus corresponding time-matched Day −1 values), with negative values set to 0.

AC02 was given as a 2 h IV infusion followed by a line flush. Peak concentrations were reached at or around the end of the infusion, with median *T*_max_ ranging from 1.00 to 2.05 h across the four dose groups. Mean terminal half-life (*t*_1/2_) ranged from 0.11 to 0.24 h. Key pharmacokinetic parameters are summarized in Table 1. AC02 exhibited approximately dose-proportional pharmacokinetics across the 0.02–0.16 mg/kg dose range. The 90% CIs for *C*_max_ and *AUC*_0–t_ were partially within the predefined 0.8–1.25 equivalence range for dose proportionality (Table 2). Mean plasma concentration-time profiles after single-ascending-dose administrations are shown in Figure 3.

#### 3.2.2. Multiple-Ascending Dose and Positive-Control Studies

In the MAD and positive-control studies, both AC02 and porcine ACTH_1-39_ reached peak plasma concentrations rapidly after administration. Median *T*_max_ ranged from 1.86 to 1.89 h for AC02 and from 1.00 to 1.50 h for porcine ACTH_1-39_. Both compounds exhibited short elimination half-lives (mean *t*_1/2_ < 0.14 h). Across the AC02 dose range of 0.04–0.08 mg/kg, both *C*_max_ and *AUC* increased with dose in a roughly dose-proportional manner. No drug accumulation was observed for AC02 or porcine ACTH_1-39_ after multiple doses. Plasma concentrations declined rapidly and fell below the lower limit of quantification (LLOQ) before the next dose. Trough concentrations on Days 3–5 were also below the LLOQ, indicating no carry-over effect from prior doses under the studied regimen. Key pharmacokinetic parameters are summarized in Supplementary Table S3, and mean plasma concentration–time profiles are presented in Figure 3d (Day 1) and Figure 3g (Day 5).

### 3.3. Pharmacodynamics

#### 3.3.1. Single-Ascending Dose Study

In the SAD study, both free and total cortisol exhibited delayed pharmacodynamic responses relative to AC02 pharmacokinetics, with median Δ*T_E_*_max_ values ranging from 2.90 to 3.34 h and 2.86 to 3.21 h, respectively. Both Δ*E*_max_ and Δ*AUEC* were significantly greater than those in the placebo group (*p*  <  0.01). However, neither parameter demonstrated dose proportionality across the evaluated dose range. Free cortisol exposure, measured by Δ*AUEC*, remained nearly identical at 0.02 and 0.04 mg/kg, and increased at 0.08 and 0.16 mg/kg. A similar trend was observed for total cortisol. Detailed results are provided in Table 3 (free cortisol) and Table 4 (total cortisol).

#### 3.3.2. Multiple-Ascending Dose and Positive-Control Studies

**Figure 4:**
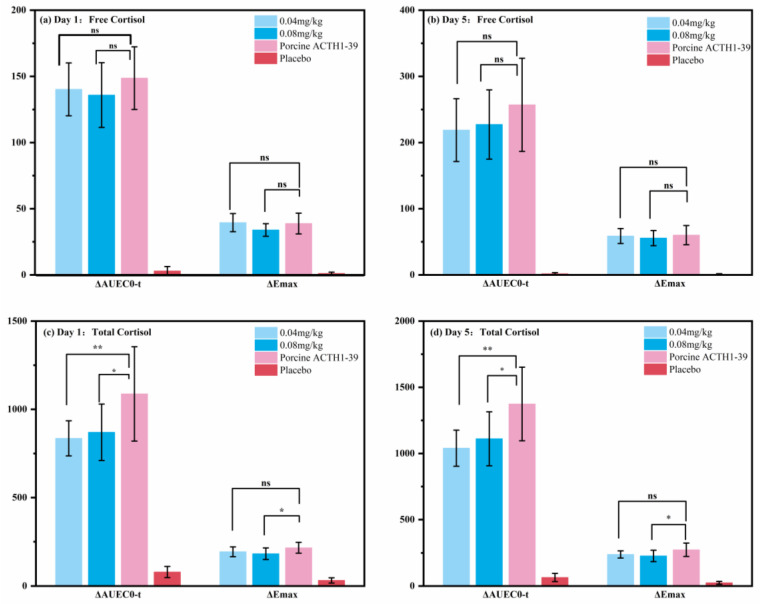
Pharmacodynamic responses of Δfree and Δtotal cortisol following repeated administration of porcine ACTH_1-39_ and AC02. Comparative bar graphs depicting PD parameters of (**a**) Δfree cortisol on Day 1, (**b**) Δfree cortisol on Day 5, (**c**) Δtotal cortisol on Day 1, and (**d**) Δtotal cortisol on Day 5 in healthy volunteers. Left panels: Δ*AUEC*_0–t_ (h·ng/mL); right panels: Δ*E*_max_ (ng/mL). Data are presented as mean ± SD (n = 10 per group). Statistical significance: ns (not significant), * (*p* < 0.05), ** (*p* < 0.01).

In the MAD study, both Δ*E*_max_ and Δ*AUEC* increased from Day 1 to Day 5 compared with placebo. Pharmacodynamic responses were compared between the 0.04 and 0.08 mg/kg AC02 cohorts and the positive-control group (porcine ACTH_1-39_) on Days 1 and 5 (Supplementary Table S4 (free cortisol) and Table S5 (total cortisol)). For free cortisol, both Δ*E*_max_ and Δ*AUEC* were similar between the two AC02 dose groups. For total cortisol, Δ*E*_max_ was comparable between the two dose groups, while Δ*AUEC*_0–14h_ showed a modest increase at 0.08 mg/kg. Comparative analysis between AC02 and the positive control (Table 5) showed that geometric mean ratios for Δ*E*_max_ and Δ*AUEC* were generally within or near the 80–125% equivalence range across both dose groups and time points. Ratios for free cortisol were closer to 100% than those for total cortisol, particularly for Δ*E*_max_ on Day 5 (93.20–98.45%). As shown in Figure 4, no statistically significant differences were observed between the AC02 dose groups and the positive control for free cortisol on Day 1 or Day 5 (*p* > 0.05). Although formal equivalence testing was not conducted, these descriptive comparisons suggest that AC02 at 0.04 and 0.08 mg/kg has PD effects generally comparable to those of the positive control, particularly for the biologically active free cortisol fraction.

### 3.4. Pharmacokinetic and Pharmacodynamic Profile

The present study characterizes the comprehensive PK and PD profiles of AC02, a novel synthetic ACTH_1-39_ analog of ACTH_1-39_, in healthy volunteers. The results demonstrate favorable PK properties for AC02, characterized by dose-proportional increases in *C*_max_ and *AUC*, a short elimination half-life, and absence of accumulation after multiple doses. These characteristics suggest a low risk of prolonged systemic exposure. Following intravenous infusion, peak plasma concentrations of AC02 were generally achieved during or immediately after the infusion period, followed by rapid clearance. Plasma concentrations fell below the LLOQ within 3 h post-dose. This kinetic profile, characterized by rapid attainment of peak concentrations followed by swift clearance, closely resembles the physiological pulsatile secretion pattern of endogenous cortisol [26,27]. In healthy individuals, cortisol is secreted in discrete pulses rather than continuously, which helps maintain the responsiveness of adrenal glands to subsequent stimulation [28]. By mimicking this natural rhythm, AC02 may preserve adrenal sensitivity more effectively than continuous hormonal exposure, which has been associated with receptor desensitization and reduced physiological responsiveness. PD analyses showed that AC02 stimulates cortisol secretion, with free and total cortisol concentrations generally increasing at higher doses, particularly at 0.16 mg/kg. The *T*_max_ for cortisol response occurred at 3.00–4.00 h, lagging behind the PK *T*_max_ and indicating a delay in signal transduction. This temporal shift aligns with established pharmacological behavior of hormone-mediated pathways, particularly those involving adrenal receptor activation and subsequent steroidogenesis [20,21].

A notable finding in this study is the dissociation between the dose-proportional PK of AC02 and the non-proportional PD responses of free cortisol. Despite an approximately 8-fold increase in *C*_max_ and *AUC* over the 0.02–0.16 mg/kg dose range, free cortisol Δ*E*_max_ increased by only about 20%, with the 0.02 and 0.04 mg/kg doses producing nearly identical responses. This pattern is consistent with receptor saturation pharmacology at the level of the adrenal MC2R receptor. Once the drug concentration reaches a level sufficient to occupy the majority of available receptors, further increases in concentration produce only marginal additional receptor activation (a classic *E*_max_ plateau effect). This interpretation is supported by the population PK/PD model, which estimated an *E*_max_ of 103.58 ng/mL for free cortisol, indicating that the cortisol stimulatory pathway approaches saturation at clinically relevant dose levels.

### 3.5. Free Cortisol Fraction

**Figure 5:**
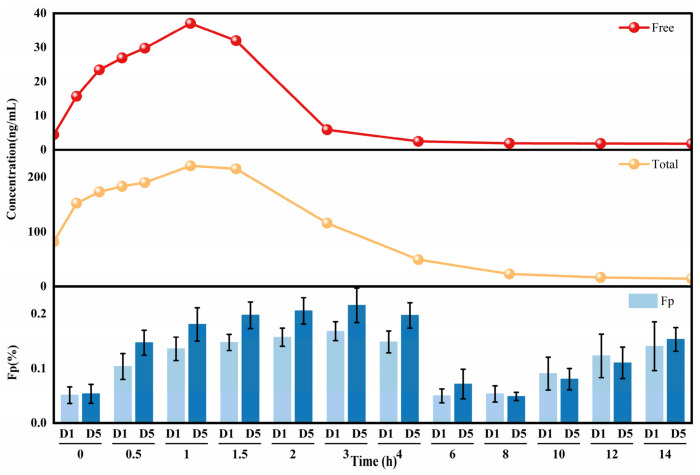
Free cortisol fraction (Fp) dynamics following 0.8 mg/kg AC02 administration on Day 1 (D1) and Day 5 (D5) in healthy volunteers. Data are presented as mean ± SD (n = 10). Fp was calculated as the ratio of free cortisol to total cortisol concentrations at each time point without baseline correction. The top panel shows the free cortisol concentration–time profiles (red: free cortisol). The middle panel shows the total cortisol concentration–time profiles (yellow: total cortisol). The bottom panel displays the corresponding Fp curve.

The free cortisol fraction (Fp) was calculated as the ratio of non-baseline-corrected free cortisol concentration to the corresponding total cortisol concentration at each timepoint. The Fp profile demonstrated an initial peak at 3 h post-dose, followed by a decline to trough levels between 6 and 8 h, and a subsequent secondary elevation observed at 10–14 h (Figure 5). This phenomenon was consistently observed across all dose groups as well as the positive control group. Integrated analysis of the free and total cortisol concentration-time profiles revealed synchronized initial peaks (3 h), followed by different temporal patterns: total cortisol exhibited a consistent monotonic decline, whereas free cortisol attained a nadir at 8 h prior to reaching a plateau phase. This kinetic difference accounts for the biphasic profile of Fp.

Through dynamic monitoring of the free cortisol fraction (Fp) over time, this study systematically elucidated a complex kinetic profile and provided key insights into the limitations of total cortisol as a PD biomarker. Across all dose groups and the positive control group, Fp consistently demonstrated a biphasic response [29]. The initial peak (occurring approximately 3 h post-dose) coincided with the rise in total cortisol, likely attributable to direct adrenocortical stimulation by AC02/ACTH via the MC2R receptor [30]. In contrast, a secondary elevation (10–14 h) emerged as a distinctive feature: while total cortisol continued to decline, free cortisol reached a nadir around 8 h before gradually recovering to form a secondary peak. Several hypotheses may explain this secondary elevation. One possible explanation is that corticosteroid-binding globulin (CBG) becomes saturated at high cortisol levels, thereby increasing the free fraction and reducing the buffering capacity for newly secreted cortisol [31]. Another hypothesis is that the hypothalamic–pituitary–adrenal (HPA) axis, after transient suppression, may undergo a rebound response that triggers additional cortisol release [32]. Both mechanisms could potentially contribute to the observed biphasic pattern, either independently or in combination. However, it must be emphasized that direct measurements of CBG, endogenous ACTH, or other adrenal biomarkers were not performed in this study; therefore, these interpretations remain speculative and should be considered hypothesis-generating rather than definitive mechanistic conclusions. The reproducibility of this biphasic pattern across all groups underscores that reliance on total cortisol alone—which exhibits a simple monophasic decay—may obscure the dynamic fluctuations of bioactive free cortisol, highlighting the limitations of total cortisol as a sole PD biomarker. Future studies incorporating direct measurements of CBG binding capacity and serial endogenous ACTH levels are warranted to test these hypotheses and further elucidate the underlying mechanisms.

### 3.6. PK/PD Modeling

Population PK/PD modeling, using an indirect response model, characterized the relationship between AC02 plasma concentrations and baseline-corrected free cortisol. The indirect response structure successfully captured the temporal delay between AC02 exposure and cortisol elevation, consistent with the known mechanism of adrenal steroidogenesis. Key fixed-effect parameter estimates are summarized in Supplementary Table S6. The estimated volume of distribution and clearance were consistent with the non-compartmental PK parameters derived from AC02 in the SAD and MAD studies (Table 1 and Table S3). The estimated *EC*_50_ was 0.55 pg/mL (approximately 0.12 pM, based on a molecular weight of ~4500 Da), suggesting that AC02 exerts its pharmacological effect at extremely low concentrations and indicating high in vivo activity. This value is consistent with the reported *EC*_50_ of 0.8 pg/mL (approximately 0.27 pM, based on a molecular weight of ~2933 Da) for synthetic ACTH_1-24_ [33], suggesting that the observed potency range is plausible for ACTH receptor agonists. However, the precision of this estimate was limited (RSE 79.08%), indicating that the concentration–response data in the low-concentration region were insufficient to support a definitive conclusion regarding potency. Several factors likely contributed to this uncertainty. First, the estimated *EC*_50_ of 0.55 pg/mL is extremely low, falling at the lower end of the concentration–response curve where data points are sparse; this region is inherently difficult to characterize precisely. Second, the steep nature of the concentration–response relationship for ACTH receptor agonists magnifies the impact of small concentration deviations on estimated *EC*_50_ values. Third, interindividual variability in baseline cortisol levels and cortisol dynamics may have further diluted the precision of *EC*_50_ estimation. Despite this limitation, the overall model structure was robust, as evidenced by the good precision (RSE < 15%) for all other parameters, including *V*, *Cl*, *E*_max_, and *K*_out_. The primary value of this model lies in its ability to describe the temporal relationship between AC02 exposure and free cortisol response, including the observed hysteresis and indirect response rather than to provide a definitive point estimate of *EC*_50_. This quantitative framework nonetheless supports dose selection for future clinical studies.

The PK/PD model presented here should be viewed primarily as a descriptive and mechanistic tool for characterizing the concentration–effect relationship of AC02 in this first-in-human study, rather than a fully qualified predictive model for prospective simulations. Formal model qualification methods were not performed, primarily due to the limited sample size (n = 52) inherent to a Phase 1 dose-escalation trial. Under such data-sparse conditions, these diagnostics tend to produce wide prediction intervals. In the present analysis, model adequacy was evaluated using standard goodness-of-fit diagnostics, which showed acceptable agreement between observed and predicted data (Supplemental Figure S1). The model structure (a one-compartment PK model with an indirect-response PD model) is mechanistically grounded and consistent with the known pharmacology of ACTH analogues. We acknowledge that the absence of formal predictive validation is a limitation of this exploratory model. Future studies with larger sample sizes will enable comprehensive qualification of the model for prospective dose optimization.

### 3.7. Immunogenicity Assessment and Safety

AC02 is a synthetic peptide with a molecular weight of 4539 Da. As with other large-molecule therapeutics, its potential to induce immune responses and generate anti-drug antibodies (ADA) was considered [34]. The formation of ADAs could theoretically influence the pharmacokinetics, pharmacodynamics, or safety of AC02, potentially leading to accelerated clearance and shortened half-life in vivo [35,36].

In the SAD study, four AC02-treated participants tested positive for ADA (pre-dose: 1/32; post-dose: 3/32, 9.4%). In the MAD study, four participants were ADA-positive, including one placebo recipient (placebo: 1/20; AC02-treated: pre-dose 1/20, post-dose 2/20). All ADA-positive samples had a titer of 1:9, and the earliest post-dose detection occurred on Day 14. Detailed immunogenicity results are presented in Supplementary Table S7. ADA-positive and ADA-negative participants showed no apparent differences in PK, PD, or safety outcomes in descriptive comparisons. However, the small number of ADA-positive cases (n = 6 in the AC02-treated group) precludes formal statistical testing, and these observations should be interpreted cautiously.

For safety, treatment-emergent adverse events (TEAEs) occurred in 11/32 (34.4%) AC02-treated subjects in the SAD study versus 4/8 (50.0%) in the placebo group; in the MAD study, TEAEs occurred in 14/20 (70.0%) AC02-treated subjects, compared versus 10/10 (100%) in the positive-control group. Most events were Grade 1 in severity, and the most frequently reported events were laboratory abnormalities, primarily elevated triglycerides. No dose-limiting toxicities or treatment-related serious adverse events were observed. Detailed safety data, including SOC/PT tabulations, are provided in Supplementary Tables S8 and S9.

Overall, AC02 was well tolerated in both the SAD and MAD studies, with no treatment-emergent serious adverse events attributed to the drug. The low-titer ADA responses observed did not show an apparent association with changes in drug exposure, pharmacodynamic effect, or safety in this small dataset.

### 3.8. Clinical Implications

A comparative analysis between AC02 and commercially available Adrenocorticotropine for Injection (porcine ACTH_1-39_) provides important insights into the therapeutic potential of this novel analog. The two drugs showed generally similar PK properties in terms of *T*_max_, elimination half-life, and steady-state behavior. However, direct comparison of systemic exposure (e.g., *C*_max_ and *AUC*) is not feasible due to fundamental differences in their origin and composition. AC02 is a fully synthetic peptide with defined structure and high purity, whereas porcine ACTH_1-39_ is derived from porcine pituitary extracts and may contain other ACTH-related peptides or degradation products that could influence its component and content. Despite these PK differences, the PD profiles were comparable. Although total cortisol levels differed between AC02 (at 0.04 and 0.08 mg/kg) and porcine ACTH_1-39_, the free cortisol Δ*E*_max_ and Δ*AUEC* values were comparable across treatments. This suggests that the biological activity of AC02 is comparable to that of porcine ACTH_1-39_, even if systemic exposure cannot be directly compared. Together with its favorable PK and safety profile, AC02 can be regarded as a viable therapeutic alternative to Adrenocorticotropine for Injection.

A key limitation of this study is that it was conducted exclusively in healthy adults, whereas the intended therapeutic population is infants with infantile spasms. The extrapolation of PK, PD, and immunogenicity data from adults to pediatric patients warrants careful consideration. First, age-related physiological differences including body weight, plasma volume, and hepatic/renal maturation may affect AC02 clearance and distribution. Second, HPA axis responsiveness in infants may differ from adults, as cortisol circadian rhythm is not fully established until early childhood, and adrenal sensitivity to ACTH may be altered in infantile spasms [37]. Third, the developing immune system in infants may exhibit different immunogenicity profiles compared with adults, which could influence ADA incidence and clinical impact. Therefore, while this first-in-human study provides essential safety, tolerability, and PK/PD data to support further clinical development, dose selection for the pediatric population should be informed by dedicated PK/PD studies in infants or by physiologically based pharmacokinetic (PBPK) modeling incorporating age-related physiological changes [38,39].

## 4. Conclusions

This present study characterizes the pharmacokinetic, pharmacodynamic, and immunogenicity profiles of AC02, a novel synthetic ACTH analog. The discovery of previously uncharacterized biphasic kinetics of the free cortisol fraction underscores the limitations of total cortisol as a sole PD biomarker and highlights the importance of assessing free cortisol dynamics in ACTH-based therapies. PK/PD modeling confirmed a clear concentration–response relationship, supporting the potent biological activity of AC02. At clinically relevant doses, AC02 demonstrated pharmacodynamic effects generally comparable to commercially available porcine ACTH_1-39_ with favorable safety. These findings provide a quantitative foundation for advancing AC02 as a therapeutic option for infantile spasms.

## Figures and Tables

**Table 1:** The pharmacokinetic parameters of AC02 in SAD study.

PK Parameters (Unit)	Mean ± SD (CV%)			
	0.02 mg/kg (n = 8)	0.04 mg/kg (n = 8)	0.08 mg/kg (n = 8)	0.16 mg/kg (n =8)
*C*_max_ (ng/mL)	48.43 ± 12.93 (26.70)	44.53 ± 2.87 (6.44)	135.90 ± 38.62 (28.42)	378.00 ± 110.44 (29.22)
* *T*_max_ (h)	1.92 (1.87, 2.12)	1.00 (1.00, 1.50)	2.05 (0.50, 2.42)	1.87 (1.82, 1.96)
*AUC*_0–t_ (ng·h/mL)	59.65 ± 8.38 (14.05)	85.40 ± 7.78 (9.10)	214.88 ± 39.75 (18.50)	469.70 ± 52.99 (11.28)
*AUC*_inf_ (ng·h/mL)	59.93 ± 8.41 (14.04)	85.61 ± 7.82 (9.14)	215.19 ± 39.79 (18.49)	470.19 ± 53.19 (11.31)
*λ*_z_ (h^−1^)	6.09 ± 1.15 (18.88)	6.42 ± 0.58 (9.09)	6.47 ± 1.36 (20.95)	4.82 ± 1.69 (35.13)
*t*_1/2_ (h)	0.12 ± 0.03 (26.28)	0.11 ± 0.01 (9.33)	0.11 ± 0.02 (20.78)	0.24 ± 0.31 (129.23)
*V*_z_ (L)	3.68 ± 0.55 (14.90)	4.88 ± 0.36 (7.42)	3.63 ± 0.65 (17.92)	6.72 ± 6.54 (97.41)
*CL* (L·h^−1^)	22.08 ± 3.97 (17.96)	31.26 ± 3.04 (9.73)	22.89 ± 3.09 (13.48)	22.84 ± 5.07 (22.22)
*MRT*_0–t_ (h)	0.32 ± 0.04 (12.85)	0.27 ± 0.02 (7.56)	0.27 ± 0.06 (20.87)	0.30 ± 0.05 (16.69)
*MRT*_inf_ (h)	0.33 ± 0.04 (12.83)	0.28 ± 0.02 (7.67)	0.27 ± 0.05 (20.37)	0.30 ± 0.06 (18.93)

n: number of replicates. SD: standard deviation. CV: coefficient of variation. * *T*_max_ is expressed as the median (min, max).

**Table 2:** Summary of statistical analysis for dose-proportionality for 0.02 mg/kg, 0.04 mg/kg, 0.08 mg/kg and 0.16 mg/kg of AC02.

PK Parameters (Unit)	Predicted Geometric Mean	Slope Estimate (90%CI)	Rdnm ^1^ (90%CI)	Conclusion ^2^
*C*_max_ (ng/mL)	(33.7, 295)	1.04 (0.89, 1.20)	1.09 (0.80, 1.50)	Approached Dose Proportionality
*AUC*_0–t_ (h·ng/mL)	(49.7, 448)	1.06 (0.96, 1.15)	1.12 (0.93, 1.37)	Approached Dose Proportionality
*AUC*_inf_ (h·ng/mL)	(49.9, 449)	1.06 (0.96, 1.15)	1.13 (0.93, 1.37)	Approached Dose Proportionality

^1^ Ratio of model-predicted mean values for high and low dose, normalized for dose. ^2^ Proportionality was concluded if the 90% confidence interval (CI) for Rdnm was contained completely within (0.8, 1.25).

**Table 3:** The pharmacodynamic parameters of free cortisol in SAD study.

PD Parameters (Unit)	Mean ± SD (CV%)				
	Placebo (n = 8)	0.02 mg/kg (n = 8)	0.04 mg/kg (n = 8)	0.08 mg/kg (n = 8)	0.16 mg/kg (n = 8)
*E*_max_ (ng/mL)	5.30 ± 4.51 (85.08)	36.80 ± 8.30 (22.56)	35.61 ± 8.90 (25.01)	42.58 ± 10.22 (24.00)	43.56 ± 14.79 (33.96)
* *T_E_*_max_ (h)	0.25 (0.00, 25.93)	2.90 (1.50, 3.12)	3.15 (2.15, 3.36)	3.11 (2.98, 3.95)	3.34 (2.81, 3.89)
*AUEC*_0–t_ (ng·h/mL)	62.91 ± 7.64 (12.14)	177.55 ± 21.98 (12.38)	189.84 ± 39.91 (21.03)	232.47 ± 43.65 (18.78)	232.37 ± 76.07 (32.74)
*AUEC*_0–14h_ (ng·h/mL)	34.90 ± 5.65 (16.19)	149.37 ± 17.82 (11.93)	157.60 ± 39.59 (25.12)	201.39 ± 42.25 (20.98)	203.94 ± 70.45 (34.54)
*AUEC*_inf_ (ng·h/mL)	312.27 ± 132.92 (42.56)	220.83 ± 52.09 (23.59)	251.83 ± 55.49 (22.03)	291.08 ± 54.00 (18.55)	285.97 ± 104.57 (36.57)
Δ*E*_max_ (ng/mL)	2.75 ± 4.30 (156.81)	33.90 ± 7.09 (20.91)	32.61 ± 8.97 (27.52)	38.88 ± 9.90 (25.47)	40.80 ± 13.95 (34.19)
* Δ*T_E_*_max_ (h)	5.05 (0.00, 25.93)	2.90 (1.50, 3.12)	3.15 (2.15, 3.36)	3.11 (2.98, 3.95)	3.34 (2.81, 3.89)
Δ*AUEC*_0–t_ (ng·h/mL)	1.08 ± 6.91 (641.43)	116.13 ± 10.60 (9.12)	117.64 ± 36.61 (31.12)	157.39 ± 39.56 (25.13)	171.48 ± 66.19 (38.60)
Δ*AUEC*_0–14h_ (ng·h/mL)	0.55 ± 1.37 (248.09)	113.97 ±1 1.97 (10.51)	117.04 ± 37.22 (31.80)	157.51 ± 38.76 (24.61)	168.79 ± 62.90 (37.26)
ΔAUEC_inf_ (ng·h/mL)	6.48 ± 7.21 (111.38)	118.11 ± 10.75 (9.10)	121.34 ± 40.33 (33.24)	159.94 ± 38.66 (24.17)	179.58 ± 73.80 (41.10)

n: number of replicates. SD: standard deviation. CV: coefficient of variation. * *T_E_*_max_ or Δ*T_E_*_max_ is expressed as the median (min, max). Δ values represent baseline-corrected concentrations (Day 1 minus time-matched Day −1 values). Negative values set to 0.

**Table 4:** The pharmacodynamic parameters of total cortisol in SAD study.

PD Parameters (Unit)	Mean ± SD (CV%)				
	Placebo (n = 8)	0.02 mg/kg (n = 8)	0.04 mg/kg (n = 8)	0.08 mg/kg (n = 8)	0.16 mg/kg (n = 8)
*E*_max_ (ng/mL)	98.85 ± 25.35 (25.65)	238.00 ± 18.90 (7.94)	229.50 ± 48.52 (21.14)	245.13 ± 35.85 (14.63)	261.38 ± 41.83 (16.00)
* *T_E_*_max_ (h)	0.00 (0.00, 25.93)	2.92 (2.87, 3.12)	3.21 (2.21, 3.36)	3.27 (2.10, 4.10)	3.81 (2.81, 3.89)
*AUEC*_0–t_ (ng·h/mL)	1044.80 ± 266.34 (25.49)	1764.88 ± 305.17 (17.29)	1700.98 ± 515.35 (30.30)	1932.19 ± 176.55 (9.14)	2248.08 ± 605.38 (26.93)
*AUEC*_0–14h_ (ng·h/mL)	591.23 ± 111.76 (18.90)	1306.55 ± 139.10 (10.65)	1358.72 ± 369.69 (27.21)	1551.20 ± 173.88 (11.21)	1762.67 ± 354.97 (20.14)
*AUEC*_inf_ (ng·h/mL)	3019.63 ± 1595.28 (52.83)	3531.13 ± 2200.54 (62.32)	2474.82 ± 1075.35 (43.45)	22,956.48 ± 38,546.17 (167.91)	6343.63 ± 8325.95 (131.25)
Δ*E*_max_ (ng/mL)	41.33 ± 24.01 (58.11)	181.50 ± 24.55 (13.52)	172.45 ± 44.83 (26.00)	187.75 ± 43.88 (23.37)	213.05 ± 37.14 (17.43)
* Δ*T_E_*_max_ (h)	6.99 (0.00, 26.23)	2.87 (1.87, 3.00)	3.21 (2.10, 4.21)	3.00 (2.10, 4.11)	2.86 (2.81, 3.87)
Δ*AUEC*_0–t_ (ng·h/mL)	35.10 ± 303.11 (863.68)	683.85 ± 237.90 (34.79)	690.35 ± 281.19 (40.73)	815.65 ± 297.91 (36.52)	1126.42 ± 518.80 (46.06)
Δ*AUEC*_0–14h_ (ng·h/mL)	−17.05 ± 78.96 (−463.07)	624.74 ± 166.60 (26.67)	748.59 ± 258.29 (34.50)	846.84 ± 270.53 (31.95)	1074.34 ± 269.32 (25.07)
Δ*AUEC*_inf_ (ng·h/mL)	265.01 ± / (/)	827.24 ± 258.33 (31.23)	878.27 ± 195.78 (22.29)	946.59 ± 363.19 (38.37)	1578.73 ± 859.62 (54.45)

n: number of replicates. SD: standard deviation. CV: coefficient of variation. * *T_E_*_max_ or Δ*T_E_*_max_ is expressed as the median (min, max). Δ values represent baseline-corrected concentrations (Day 1 minus time-matched Day −1 values). Negative values set to 0.

**Table 5:** The PD effects of AC02 (0.04 mg/kg and 0.08 mg/kg) compared with the positive-control drug.

Day	Analyte	PD Parameters	Geomean			%Ratio (90%CI)	
			0.04 mg/kg	0.08 mg/kg	Positive-Control	0.04 mg/kg/ Positive-Control	0.08 mg/kg/ Positive-Control
Day 1	Free cortisol	Δ*E*_max_ (ng/mL)	38.97	33.66	38.12	102.22 (89.93–116.20)	88.29 (77.67–100.36)
	Free cortisol	Δ*AUEC*_0–14h_ (ng·h/mL)	133.63	128.61	149.94	89.13 (78.67–100.98)	85.77 (75.71–97.18)
	Total cortisol	Δ*E*_max_ (ng/mL)	192.43	180.32	214.38	89.76 (79.77–101.02)	84.12 (74.75–94.66)
	Total cortisol	Δ*AUEC*_0–14h_ (ng·h/mL)	763.17	782.00	1023.42	74.57 (61.77–90.03)	76.41 (63.29–92.25)
Day 5	Free cortisol	Δ*E*_max,ss_ (ng/mL)	57.74	54.67	58.65	98.45 (84.10–115.24)	93.20 (79.62–109.11)
	Free cortisol	Δ*AUEC*_0–14h,ss_ (ng·h/mL)	204.91	216.23	258.58	79.24 (66.69–94.16)	83.62 (70.38–99.35)
	Total cortisol	Δ*E*_max,ss_ (ng/mL)	237.21	224.10	269.52	88.01 (77.73–99.66)	83.15 (73.43–94.15)
	Total cortisol	Δ*AUEC*_0–14h,ss_ (ng·h/mL)	942.48	1028.45	1361.08	69.24 (59.70–80.31)	75.56 (65.15–87.64)

## Data Availability

Data presented in this study is contained within the article and Supplementary Material. Further inquiries can be directed to the corresponding authors.

## Supplementary Materials

The following supporting information can be downloaded at: https://www.mdpi.com/article/10.3390/pharmaceutics18070860/s1, Figure S1. Goodness-of-Fit Diagnostics for the Population PK/PD Model of AC02. (A) Observed vs. population-predicted plasma concentrations of AC02. (B) Observed vs. individual-predicted plasma concentrations of AC02. (C) Observed vs. population-predicted baseline-adjusted free cortisol concentrations. (D) Observed vs. individual-predicted baseline-adjusted free cortisol concentrations. The solid line represents the line of identity (*y* = *x*). Table S1. Calibration range and mass spectrum parameters of AC02, porcine ACTH_1-39_, free and total cortisol. Table S2. Summary of demographic characteristics. Table S3. The pharmacokinetic parameters of AC02 and porcine ACTH_1-39_ in MAD study on day 1 and day 5. Table S4. The pharmacodynamic parameters of free cortisol in MAD study on day 1 and day 5. Table S5. The pharmacodynamic parameters of total cortisol in MAD study on day 1 and day 5. Table S6. Population PK/PD parameter estimates of AC02 effects on baseline-adjusted free cortisol. Table S7. Immunogenicity assessment results of AC02. Table S8. Summary of safety events. Table S9. Treatment-emergent adverse events by system organ class and preferred term (≥5% in any group).
