# Supplementary material for: Pharmacokinetics, Pharmacodynamics and Immunogenicity of AC02, a Novel Synthetic Derivate Peptide of Human Adrenocorticotropic Hormone for Infantile Spasms
Source: Pharmaceutics. 2026 Jul 14;18(7):860. doi: 10.3390/pharmaceutics18070860 (PMC13414577; doi:10.3390/pharmaceutics18070860)
Supplement: Supplementary file 1 [file pharmaceutics-18-00860-s001.zip › pharmaceutics-4382554-supplementary.pdf]

Supplementary Information

Pharmacokinetics, Pharmacodynamics and Immunogenicity of AC02, a Novel Synthetic Derivate Peptide of Human Adreno-corticotropic Hormone for Infantile Spasms

Shunbo Zhao [1, 2], Bingda Wu [1], Hui Shen [2], Qi Zhou [2], Minlu Cheng [1, 2], Chang Shu [1*], Li Ding [1, 2, 3*]

[1] Department of Pharmaceutical Analysis, School of Pharmacy, China Pharmaceutical University, Nan-jing 211198, China

[2] Nanjing Clinical Tech Laboratories Inc., 18 Zhilan Road, Jiangning District, Nanjing 211100, China

[3.] Nanjing Jiening Pharmaceutical Technology, 18 Zhilan Road, Jiangning District, Nanjing 211100, China

[*] Correspondence author E-mail addresses:

dingli@cpu.edu.cn (Li Ding)

shuchang@cpu.edu.cn (Chang Shu)

TABLE OF CONTENTS

Figures: Figure S1

Figure S1 Goodness-of-Fit Diagnostics for the Population PK/PD Model of AC02. (*Page S2*)

Tables: Table S1-9

Table S1 Calibration range and mass spectrum parameters of AC02, porcine $ACTH_{1-39}$, free and total cortisol (*Page S3*)

Table S2 Summary of demographic characteristics. (*Page S3*)

Table S3 The pharmacokinetic parameters of AC02 and porcine $ACTH_{1-39}$ in MAD study on day 1 and day 5. (*Page S4-5*)

Table S4 The pharmacodynamic parameters of free cortisol in MAD study on day 1 and day 5. (*Page S6-7*)

Table S5 The pharmacodynamic parameters of total cortisol in MAD study on day 1 and day 5. (*Page S8-9*)

Table S6 Population PK/PD parameter estimates of AC02 effects on baseline-adjusted free cortisol

(*Page S9*)

Table S7 Immunogenicity assessment results of AC02 (*Page S10*)

Table S8. Summary of safety events (*Page S11*)

Table S9. Treatment-emergent adverse events by system organ class and preferred term (⩾5% in any group) (*Page S12*)

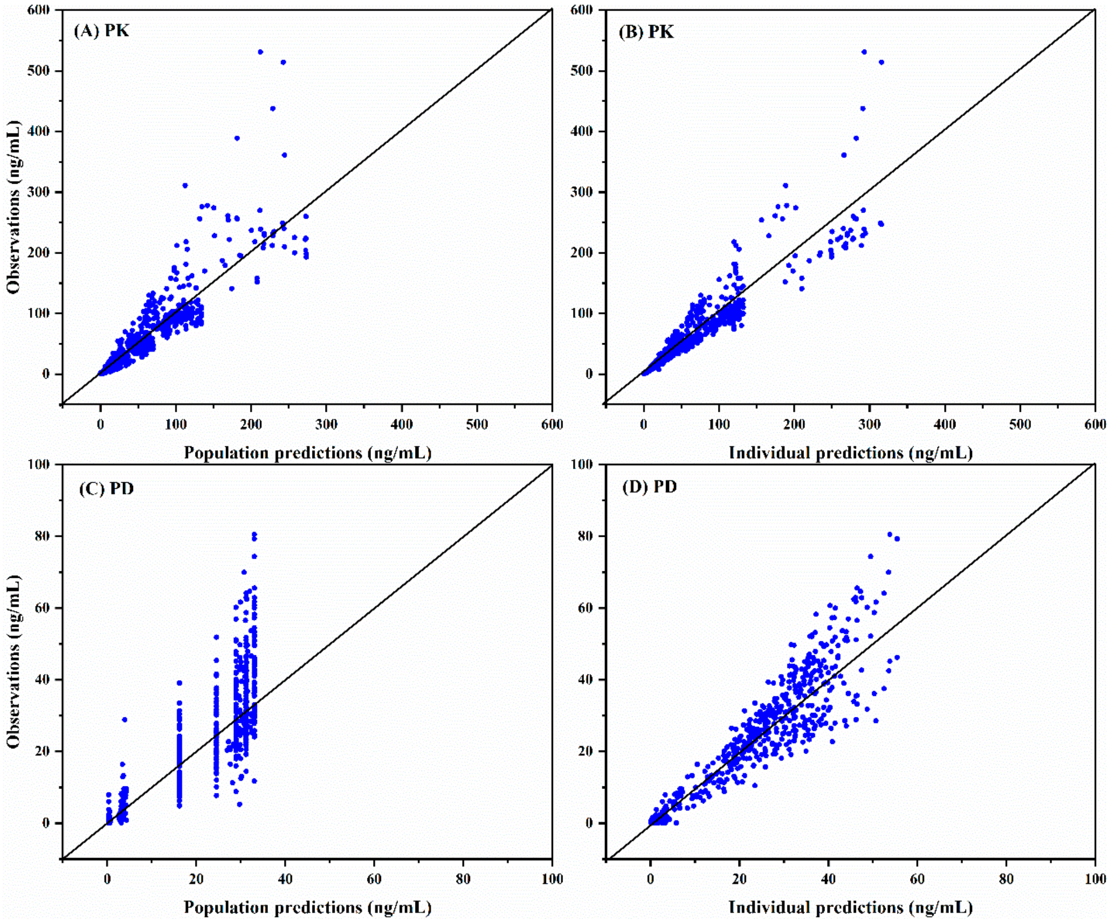

Figure S1 Goodness-of-Fit Diagnostics for the Population PK/PD Model of AC02. (A) Observed vs. population-predicted plasma concentrations of AC02. (B) Observed vs. individual-predicted plasma concentrations of AC02. (C) Observed vs. population-predicted baseline-adjusted free cortisol concentrations. (D) Observed vs. individual-predicted baseline-adjusted free cortisol concentrations. The solid line represents the line of identity ($y = x$).

Table S1 Calibration range and mass spectrum parameters of AC02, porcine $ACTH_{1-39}$, free and total cortisol.

Analyte	Calibration range	Precursor ion (*m/z*)	Product ion (*m/z*)	DP (V)	EP (V)	CE (V)	CXP (V)
AC02	0.500 – 200 ng/mL	757.6 $[M+6H]^{6+}$	876.0	65	10	24	20
Porcine $ACTH_{1-39}$	0.100 – 10.0 ng/mL	762.2$[M+6H]^{6+}$	881.3	65	10	24	20
Des-13Val (IS)	-	741.4$[M+6H]^{6+}$	856.4	65	10	24	20
Free Cortisol	0.500 – 500 ng/mL	363.2$[M+H]^{+}$	121.2	80	10	32	6
Total Cortisol	5.00 – 1000 ng/mL	363.2$[M+H]^{+}$	121.2	80	10	32	6
Cortisol-D_4 (IS)	-	367.2$[M+H]^{+}$	121.2	80	10	32	6

Abbreviation: DP, Declustering Potential; EP, Entrance Potential; CE, Collision Energy; CXP, Collision Cell Exit Potential.

Table S2 Summary of demographic characteristics.

Group	SAD (Mean±SD)					MAD (Mean±SD)			Positive-control (n=10)
	0.02 mg/kg (n=8)	0.04 mg/kg (n=8)	0.08 mg/kg (n=8)	0.16 mg/kg (n=8)	Placebo (n=8)	0.04 mg/kg (n=10)	0.08 mg/kg (n=10)	Placebo (n=4)	
Male, n (%)	7(87.5)	5(62.5)	6(75.0)	7(87.5)	8(100.0)	9(90.0)	8(80.0)	3(75.0)	8(80.0)
Female, n (%)	1(12.5)	3(37.5)	2(25.0)	1(12.5)	0(0.0)	1(10.0)	2(20.0)	1(25.0)	2(20.0)
Age (y)	31.75±7.96	29.63±6.30	26.88±7.12	31.25±7.40	33.75±6.20	37.20±4.57	32.50±7.56	36.00±4.97	35.50±4.55
Weight (kg)	65.01±9.45	66.60±6.62	60.58±7.98	65.81±8.74	64.16±7.33	69.32±6.57	63.42±7.92	67.70±6.61	64.70±4.41
BMI (kg/m^2)	21.42±1.86	22.81±1.58	21.43±2.01	23.16±1.92	22.35±1.39	23.20±1.70	22.53±1.70	22.80±2.17	22.80±1.31

BMI indicates body mass index.

Table S3 The pharmacokinetic parameters of AC02 and porcine $ACTH_{1-39}$ in MAD study on day 1 and day 5.

PK Parameters(unit)	Day 1			Day 5		
	0.04 mg/kg (n=10)	0.08 mg/kg (n=10)	Positive-control(n=10)	0.04 mg/kg (n=10)	0.08 mg/kg (n=10)	Positive-control(n=10)
C_{max}(ng/mL)/ $C_{max,ss}$(ng/mL)	76.88±26.51(34.48)	140.84±46.80(33.23)	3.99±0.59(14.68)	90.59±22.73(25.09)	142.96±34.85(24.38)	3.38±0.67(19.79)
$C_{min,ss}$(ng/mL)	/	/	/	0.00±0.00(/)	0.00±0.00(/)	0.00±0.00(/)
$C_{av,ss}$(ng/mL)	/	/	/	4.23±0.57(13.40)	7.94±1.15(14.50)	0.27±0.04(16.48)
*T_{max}(h)/$T_{max,ss}$(h)	1.86(1.00,2.13)	1.88(0.50,1.97)	1.00(0.51,2.22)	1.87(1.00,1.94)	1.89(1.83,1.99)	1.50(0.50,2.27)
$AUC_{0\text{-}t}$(ng·h/mL) /$AUC_{0\text{-}t,ss}$(ng·h/mL)	107.58±16.01(14.89)	199.57±27.17(13.62)	7.31±0.97(13.27)	101.15±13.57(13.41)	190.07±27.54(14.49)	6.53±1.08(16.59)
$AUC_{0\text{-}24h}$(ng·h/mL) /$AUC_{0\text{-}tau,ss}$(ng·h/mL)	108.07±15.95(14.75)	199.78±27.09(13.56)	7.37±0.99(13.47)	101.53±13.61(13.40)	190.62±27.64(14.50)	6.55±1.08(16.48)
AUC_{inf}(ng·h/mL) /$AUC_{inf,,ss}$(ng·h/mL)	108.07±15.95(14.75)	199.78±27.09(13.56)	7.37±0.99(13.47)	101.53±13.61(13.40)	190.62±27.64(14.50)	6.55±1.08(16.48)
λ_z(h^{-1})/$\lambda_{z,ss}$(h^{-1})	6.24±0.55(8.74)	5.69±0.82(14.39)	5.02±0.81(16.06)	6.79±0.45(6.66)	6.38±0.89(13.95)	5.19±0.95(18.37)
$t_{1/2}$(h)/$t_{1/2,ss}$(h)	0.11±0.01(9.26)	0.12±0.02(12.34)	0.14±0.02(16.01)	0.10±0.01(6.02)	0.11±0.02(14.63)	0.14±0.02(16.45)
#V_z(L)/V_{ss}(L)	4.17±0.46(11.05)	4.54±0.52(11.47)	0.0007±0.0002(25.12)	4.07±0.42(10.41)	4.27±0.60(14.08)	0.0008±0.0002 (25.82)
#CL(L·h^{-1})/CL_{ss}(L·h^{-1})	26.05±3.70(14.21)	25.77±4.08(15.84)	0.0035±0.0005(14.55)	27.55±2.29(8.33)	27.06±4.42(16.32)	0.0039±0.0007 (17.37)
$MRT_{0\text{-}t}$(h)	0.26±0.03(10.36)	0.26±0.05(18.34)	0.30±0.04(13.29)	/	/	/
MRT_{inf}(h)/$MRT_{inf,ss}$(h)	0.27±0.03(9.70)	0.26±0.05(17.93)	0.31±0.04(13.38)	0.29±0.03(11.37)	0.27±0.03(9.42)	0.30±0.04(14.45)
DF(%)	/	/	/	2118.32±382.18(18.04)	1793.04±323.15(18.02)	1231.77±60.78(4.93)
R_{ac}(C_{max})	/	/	/	1.24±0.38(30.83)	1.07±0.30(27.71)	0.84±0.07(7.93)
R_{ac}(AUC)	/	/	/	0.94±0.09(9.34)	0.96±0.08(7.88)	0.89±0.07(7.74)

Abbreviation: n, number of replicates. SD, standard deviation. CV, coefficient of variation. *T_{max} or $T_{max,ss}$ is expressed as the median (min, max). R_{ac}(C_{max}) and R_{ac}(AUC) are calculated by dividing the AUC and C_{max} during the dosing interval on Day 5 by their respective values during the dosing interval on Day 1. #The unit of V_z/V_{ss} for the positive-control drug is U/(ng/L), and the unit of CL/CL_{ss} is U/(h·ng/L).

Table S4 The pharmacodynamic parameters of free cortisol in MAD study on day 1 and day 5.

PD Parameters(unit)	Day 1				Day 5			
	Placebo (n=4)	0.04 mg/kg (n=10)	0.08 mg/kg (n=10)	Positive-control (n=10)	Placebo (n=4)	0.04 mg/kg (n=10)	0.08 mg/kg (n=10)	Positive-control (n=10)
E_{max}(ng/mL)/ $E_{max,ss}$(ng/mL)	3.93±0.66 (16.92)	42.37±7.20 (17.00)	37.03±4.43 (11.95)	41.73±7.83 (18.76)	3.47±0.72 (20.80)	61.59±11.91 (19.34)	58.76±10.90 (18.54)	63.20±14.40 (22.78)
$E_{min,ss}$(ng/mL)	/	/	/	/	1.71±0.31 (18.39)	1.82±0.27 (14.87)	1.48±0.19 (12.77)	2.55±0.52 (20.31)
$E_{av,ss}$(ng/mL)	/	/	/	/	2.19±0.32 (14.65)	11.23±1.98 (17.64)	11.38±2.10 (18.44)	22.61±4.90 (21.67)
*T_{Emax}(h)/$T_{Emax,ss}$(h)	0.89 (0.00,2.98)	2.86 (2.84,3.13)	2.86 (2.77,2.90)	3.10 (2.02,3.23)	1.68 (0.00,2.87)	2.87 (2.78,2.94)	2.89 (2.83,3.92)	3.15 (2.20,3.26)
$AUEC_{0-t}$(ng·h/mL)/ $AUEC_{0-t,ss}$(ng·h/mL)	34.78±5.80 (16.69)	174.71±22.34 (12.79)	163.53±23.82 (14.57)	195.71±26.98 (13.78)	56.94±8.41 (14.77)	273.88±47.58 (17.37)	277.33±50.60 (18.25)	651.49±142.01 (21.80)
$AUEC_{0-14h}$(ng·h/mL)/ $AUEC_{0-14h,ss}$(ng·h/mL)	35.04±5.67 (16.18)	174.94±22.26 (12.72)	163.77±23.78 (14.52)	195.34±26.93 (13.79)	32.19±4.20 (13.06)	249.25±47.85 (19.20)	254.38±49.79 (19.58)	309.64±73.84 (23.85)
$AUEC_{0-tau,ss}$(ng·h/mL)	/	/	/	/	52.58±7.70 (14.65)	269.62±47.57 (17.64)	273.14±50.36 (18.44)	542.76±117.63 (21.67)
$AUEC_{inf}$(ng·h/mL)/ $AUEC_{inf,ss}$(ng·h/mL)	65.25±22.05 (33.79)	199.22±34.00 (17.07)	215.61±82.61 (38.31)	219.87±28.69 (13.05)	475.27±140.31 (29.52)	331.97±79.87 (24.06)	306.18±52.80 (17.25)	4872.80±1873.06 (38.44)
ΔE_{max}(ng/mL)/ $\Delta E_{max,ss}$(ng/mL)	1.06±1.03 (97.51)	39.50±6.82 (17.28)	33.92±4.76 (14.02)	38.80±7.85 (20.25)	0.94±0.86(92.21)	58.72±11.34 (19.32)	55.64±11.48 (20.64)	60.13±14.47 (24.07)
$\Delta E_{min,ss}$(ng/mL)	/	/	/	/	-2.38±1.54 (-64.84)	-2.38±1.82 (-76.53)	-1.03±0.52 (-50.95)	-1.85±2.58 (-139.61)

PD Parameters(unit)	Day 1<br>Placebo (n=4)	Day 1<br>0.04 mg/kg (n=10)	Day 1<br>0.08 mg/kg (n=10)	Day 1<br>Positive-control (n=10)	Day 5<br>Placebo (n=4)	Day 5<br>0.04 mg/kg (n=10)	Day 5<br>0.08 mg/kg (n=10)	Day 5<br>Positive-control (n=10)
$\Delta E_{av,ss}$(ng/mL)	/	/	/	/	-0.21±0.24<br>(-111.52)	8.48±1.97<br>(23.19)	9.13±2.18<br>(23.88)	19.58±4.81<br>(24.57)
*ΔT_{Emax}(h)/<br>$\Delta T_{Emax,ss}$(h)	2.40<br>(0.50,7.81)	2.86<br>(2.84,3.13)	2.86<br>(2.77,2.90)	3.10<br>(2.02,3.23)	2.86<br>(2.76,2.90)	2.87<br>(2.78,2.94)	2.89<br>(2.83,3.92)	3.15<br>(2.20,3.26)
$\Delta AUEC_{0\text{-}t}$(ng·h/mL)/<br>$\Delta AUEC_{0\text{-}t,ss}$(ng·h/mL)	-0.78±1.74<br>(-222.22)	135.15±20.80<br>(15.39)	130.54±25.45<br>(19.49)	151.61±25.35<br>(16.72)	-5.48±6.75<br>(-123.13)	202.60±47.34<br>(23.36)	219.05±52.58<br>(24.00)	572.02±139.43<br>(24.38)
$\Delta AUEC_{0\text{-}14h}$(ng·h/mL)/<br>$\Delta AUEC_{0\text{-}14h,ss}$(ng·h/mL)	0.58±1.05<br>(181.91)	135.36±22.06<br>(16.30)	135.56±29.20<br>(21.54)	151.62±25.36<br>(16.73)	-3.64±2.67<br>(-73.37)	209.44±46.06<br>(21.99)	221.11±51.43<br>(23.26)	265.92±73.07<br>(27.48)
$\Delta AUEC_{0\text{-}tau,ss}$(ng·h/mL)	/	/	/	/	-5.09±5.68<br>(-111.52)	203.52±47.19<br>(23.19)	219.23±52.34<br>(23.88)	469.90±115.46<br>(24.57)
$\Delta AUEC_{inf}$(ng·h/mL)/<br>$\Delta AUEC_{inf,ss}$(ng·h/mL)	1.49±1.57<br>(105.21)	131.89±19.11<br>(14.49)	136.17±29.06<br>(21.34)	152.89±25.36<br>(16.59)	-5.55±/(/)	222.01±51.69<br>(23.28)	237.53±57.26<br>(24.11)	2039.08±683.78<br>(33.53)

Abbreviation: n, number of replicates. SD, standard deviation. CV, coefficient of variation. *T_{Emax} or $T_{Emax,ss}$ is expressed as the median (min, max). Δ values represent baseline-corrected concentrations (Day 1/Day 5 minus time-matched Day -1 values); negative values set to 0.

Table S5 The pharmacodynamic parameters of total cortisol in MAD study on day 1 and day 5.

PD Parameters(unit)	Day 1				Day 5			
	Placebo (n=4)	0.04 mg/kg (n=10)	0.08 mg/kg (n=10)	Positive-control (n=10)	Placebo (n=4)	0.04 mg/kg (n=10)	0.08 mg/kg (n=10)	Positive-control (n=10)
E_{max}(ng/mL)/ $E_{max,ss}$(ng/mL)	79.05±11.47 (14.51)	232.50±32.19 (13.85)	222.10±23.94 (10.78)	265.50±23.67 (8.91)	77.30±14.97 (19.36)	279.50±26.99 (9.66)	271.30±29.25 (10.78)	323.20±43.90 (13.58)
$E_{min,ss}$(ng/mL)	/	/	/	/	12.08±4.58 (37.89)	10.77±2.55 (23.71)	10.44±1.82 (17.42)	13.29±5.30 (39.91)
$E_{av,ss}$(ng/mL)	/	/	/	/	30.73±8.63 (28.07)	71.43±8.97 (12.55)	76.15±8.54 (11.21)	135.05±19.30 (14.29)
*T_{Emax}(h)/$T_{Emax,ss}$(h)	0.89 (0.00,5.98)	2.86 (2.84,3.13)	2.89 (2.77,3.88)	3.20 (2.13,4.09)	1.43 (0.00,2.87)	2.87 (1.50,2.94)	2.92 (2.83,3.90)	3.23 (3.10,4.08)
$AUEC_{0-t}$(ng·h/mL)/ $AUEC_{0-t,ss}$(ng·h/mL)	552.05±83.14 (15.06)	1331.45±159.73 (12.00)	1357.17±172.36 (12.70)	1716.30±287.41 (16.75)	807.00±236.97 (29.36)	1770.58±226.06 (12.77)	1895.03±215.83 (11.39)	3809.02±533.80 (14.01)
$AUEC_{0-14h}$(ng·h/mL)/ $AUEC_{0-14h,ss}$(ng·h/mL)	554.02±83.42 (15.06)	1333.39±159.24 (11.94)	1358.87±172.19 (12.67)	1713.77±286.87 (16.74)	494.79±101.63 (20.54)	1513.98±190.86 (12.61)	1606.35±196.71 (12.25)	2036.84±312.56 (15.35)
$AUEC_{0-tau,ss}$(ng·h/mL)	/	/	/	/	737.55±207.06 (28.07)	1714.33±215.18 (12.55)	1827.61±204.90 (11.21)	3241.14±463.22 (14.29)
$AUEC_{inf}$(ng·h/mL)/ $AUEC_{inf,ss}$(ng·h/mL)	598.09±87.02 (14.55)	1493.76±247.88 (16.59)	1416.99±172.24 (12.16)	1798.76±299.35 (16.64)	5618.15±7127.65 (126.87)	2171.60±425.24 (19.58)	2617.31±820.07 (31.33)	70770.47±46641.58 (65.91)
ΔE_{max}(ng/mL)/ $\Delta E_{max,ss}$(ng/mL)	31.70±14.79 (46.66)	194.23±27.81 (14.32)	182.92±32.87 (17.97)	216.25±30.12 (13.93)	24.35±10.55 (43.31)	238.57±27.14 (11.37)	227.51±42.79 (18.81)	273.43±49.85 (18.23)
$\Delta E_{min,ss}$(ng/mL)	/	/	/	/	-51.23±19.27 (-37.62)	-38.47±18.96 (-49.29)	-28.29±18.83 (-66.56)	-36.25±28.66 (-79.07)
$\Delta E_{av,ss}$(ng/mL)	/	/	/	/	-4.10±6.10 (-148.76)	35.12±10.51 (29.92)	41.56±11.05 (26.59)	93.95±20.59 (21.91)
*ΔT_{Emax}(h)/ $\Delta T_{Emax,ss}$(h)	7.88 (1.81,9.81)	2.86 (1.86,3.13)	3.79 (1.50,3.90)	3.10 (2.13,4.09)	2.86 (2.76,2.90)	2.86 (1.50,2.94)	3.86 (2.83,3.95)	3.27 (2.22,4.27)

PD Parameters(unit)	Day 1				Day 5			
	Placebo (n=4)	0.04 mg/kg (n=10)	0.08 mg/kg (n=10)	Positive-control (n=10)	Placebo (n=4)	0.04 mg/kg (n=10)	0.08 mg/kg (n=10)	Positive-control (n=10)
$\Delta AUEC_{0\text{-}t}$(ng·h/mL)/ $\Delta AUEC_{0\text{-}t,ss}$(ng·h/mL)	-12.48±54.93 (-440.16)	772.67±128.71 (16.66)	803.50±185.57 (23.10)	1065.72±317.54 (29.80)	-107.90±179.77 (-166.62)	822.89±270.15 (32.83)	981.59±289.26 (29.47)	2712.22±576.40 (21.25)
$\Delta AUEC_{0\text{-}14h}$(ng·h/mL)/ $\Delta AUEC_{0\text{-}14h,ss}$(ng·h/mL)	/	754.75±122.00 (16.16)	786.97±209.01 (26.56)	1065.53±317.13 (29.76)	-71.86±81.28 (-113.11)	954.03±154.15 (16.16)	1050.40±227.57 (21.66)	1386.28±291.35 (21.02)
$\Delta AUEC_{0\text{-}tau,ss}$(ng·h/mL)	/	/	/	/	-98.35±146.30 (-148.76)	842.81±252.15 (29.92)	997.40±265.19 (26.59)	2254.83±494.10 (21.91)
$\Delta AUEC_{inf}$(ng·h/mL)/ $\Delta AUEC_{inf,ss}$(ng·h/mL)	/	794.19±104.94 (13.21)	821.48±231.07 (28.13)	1145.92±334.47 (29.19)	-92.12±60.37 (-65.54)	1024.49±324.12 (31.64)	1208.21±344.87 (28.54)	80075.18±4663.32 (5.82)

Table S6 Population PK/PD parameter estimates of AC02 effects on baseline-adjusted free cortisol.

**Parameter**	**Typical Values (RSE%)**	**Shrinkage (%)**
V(L)	3.92(2.96)	13.11
Cl(L/h)	22.48(3.53)	2.9
R_0	0.32(14.73)	21.62
K_{out}(1/h)	1.29(4.71)	39.92
E_{max}(ng/mL)	103.58(14.89)	28.18
EC_{50}(pg/mL)	0.55(79.08)	26.67

Table S7 Immunogenicity assessment results of AC02.

Group	Dose cohort	Subject ID	Pre-dose	Day 14	Day 28	Titer
SAD	0.02 mg/kg (n = 8)	S0404	+	−	−	1:9
	0.04 mg/kg (n = 8)	S0102	−	−	+	1:9
		S0103	−	+	−	1:9
		S0107	−	+	−	1:9
MAD	Placebo	M0202	−	+	−	1:9
	0.04 mg/kg (n = 10)	M0104	−	+	−	1:9
	0.08 mg/kg (n = 10)	M0206	−	−	+	1:9
		M0207	+	−	−	1:9

Note: "+" represented ADA-positive result, and "-" represented ADA-negative result.

Table S8. Summary of safety events.

Parameter	SAD<br>AC02 (n=32)	SAD<br>Placebo (n=8)	MAD<br>AC02 (n=20)	MAD<br>Placebo (n=4)	MAD<br>Positive control (n=10)
**Adverse events (AEs)**					
Subjects with any AE, n (%)	11 (34.4)	4 (50.0)	14 (70.0)	1 (25.0)	10 (100)
**Adverse reactions (ADRs)**					
Subjects with any ADR, n (%)	8 (25.0)	4 (50.0)	13 (65.0)	1 (25.0)	7 (70.0)
**Severity**					
Grade 1 (mild)	10 (31.3)	3 (37.5)	11 (55.0)	0	8 (80.0)
Grade 2 (moderate)	1 (3.1)	1 (12.5)	2 (10.0)	1 (25.0)	2 (20.0)
Grade ≥3 (severe)	1 (3.1)*	0	0	0	0
**Serious AEs (SAEs)**	1 (3.1)*	0	0	0	0
**Discontinuation due to AEs**	0	0	0	0	0

* Rib fracture, assessed as unlikely related to study drug.

Table S9. Treatment-emergent adverse events by system organ class and preferred term (≥5% in any group).

SOC / PT	SAD<br>AC02 (n=32)	SAD<br>Placebo (n=8)	MAD<br>AC02 (n=20)	MAD<br>Placebo (n=4)	MAD<br>Positive control (n=10)
**Investigations**	**9 (28.1)**	**4 (50.0)**	**11 (55.0)**	**1 (25.0)**	**7 (70.0)**
Blood triglycerides increased	3 (9.4)	3 (37.5)	9 (45.0)	1 (25.0)	1 (10.0)
White blood cells urine positive	2 (6.3)	0	—	—	—
Blood fibrinogen decreased	0	0	2 (10.0)	0	0
**Gastrointestinal disorders**	**1 (3.1)**	**0**	**1 (5.0)**	**0**	**4 (40.0)**
Abdominal pain	0	0	0	0	3 (30.0)
Diarrhea	0	0	1 (5.0)	0	3 (30.0)
**Psychiatric disorders**	**0**	**0**	**1 (5.0)**	**0**	**1 (10.0)**
**Blood and lymphatic system disorders**	**0**	**0**	**1 (5.0)**	**0**	**1 (10.0)**

Note: Only SOCs/PTs with ≥5% incidence in any group are shown.
